# Evaluation of the therapeutic effect of *Descurainia sophia* (L.) Webb ex Prantl seed extract on hyperthyroidism: A double‐blind placebo‐controlled pilot clinical trial

**DOI:** 10.1002/fsn3.3522

**Published:** 2023-07-04

**Authors:** Fatemeh Farzameh, Mohammad Azadbakht, Zahra Kashi, Hossein Asgarirad, Ebrahim Salehifar, Fatemeh Mirzaee, Ali Davoodi, Shervin Amirkhanloo

**Affiliations:** ^1^ Department of Pharmacognosy and Biotechnology, Faculty of Pharmacy Mazandaran University of Medical Sciences Sari Iran; ^2^ Diabetes Research Center Mazandaran University of Medical Sciences Sari Iran; ^3^ Department of Pharmaceutics, Faculty of Pharmacy Mazandaran University of Medical Sciences Sari Iran; ^4^ Pharmaceutical Sciences Research Center, Hemoglobinopathy Institute Mazandaran University of Medical Sciences Sari Iran; ^5^ Medicinal Plants Research Center Mazandaran University of Medical Sciences Sari Iran; ^6^ Medicinal Plants Research Center Mazandaran University of Medical Sciences Sari Iran

**Keywords:** *Descurainia sophia*, hyperthyroidism, khaksheer, thyrotoxicosis

## Abstract

The seeds of *Descurainia sophia* (L.) Webb ex Prantl contain goitrogenic glucosinolates, such as gluconapin (3‐butenyl glucosinolate). Because of the important role of iodine in the synthesis of thyroid hormones and the inhibitory activity of *D. sophia* on iodine uptake by the thyroid gland, this study aimed to determine the effects of *D. sophia* syrup on clinical and biochemical variables of thyrotoxicosis in hyperthyroid patients. In this randomized, double‐blind trial, 10 newly diagnosed hyperthyroid female patients were randomly assigned to treatment with (1) methimazole (MMI) plus *D. sophia* capsules (350 mg/d) or (2) MMI plus placebo capsules. The primary outcomes were clinical and biochemical manifestations of hyperthyroidism after 60 days. Serum levels of FT3 and FT4 significantly decreased (5.9 ± 2.5 vs. 9.4 ± 5.9 and 29.1 ± 3.1 vs. 31.77 ± 3.7, respectively) and the serum thyroid‐stimulating hormone (TSH) concentration significantly increased in the *D. sophia* group contrasted to the placebo group (4.7 ± 0.1 vs. 0.05 ± 0.02). Significant improvement in the thyrotoxicosis clinical symptoms was reported in the *D. sophia* group contrasted to the placebo group (*p* < .05). *D. sophia* can suppress plasma levels of FT3 and FT4 and may be useful as adjunctive therapy for hyperthyroidism.

## Graphical abstract

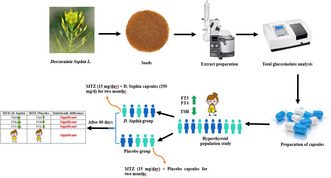

## 1 INTRODUCTION

Hyperthyroidism is characterized by enlarged synthesis and secretion of thyroid hormones (T4 and T3) from the thyroid gland. Thyroid hormones are essential for normal growth, reproduction, and the regulation of energy metabolism. Common symptoms of hyperthyroidism include palpitations, tremor, fatigue, anxiety, sleep disorders, weight loss, heat intolerance, and sweating. Frequent physical findings include tachycardia, tremors, and weight loss (Boelaert et al., 2010).

Graves' disease (GD), which is the most common type of hyperthyroidism (Yang et al., 2002), is an autoimmune disease related to the humoral immune response. Other important causes of hyperthyroidism include toxic adenoma and drugs, such as amiodarone. Hyperthyroidism is a common condition with potentially adverse health consequences that can affect all populations around the world. The prevalence of hyperthyroidism in areas with adequate iodine intake ranges from 0.2% to 1.3% (Taylor et al., 2018). Despite increasing awareness of thyroid disease and the availability of sensitive laboratory tests to measure thyroid hormones, cases of severe thyroid dysfunction still occur occasionally (Rice et al., 2013; Taylor et al., 2015).

Undiagnosed or untreated hyperthyroidism can have detrimental health effects (De Leo et al., 2016). The treatment of hyperthyroidism involves symptom relief and therapy with antithyroid drugs, radioactive iodine therapy, and surgery. These treatments have some advantages and disadvantages (Ross et al., 2016), first of all, patients need to use drugs for a long time and also we see the recurrence of the disease after treatment (Yoshihara et al., 2014). The adverse effects of methimazole (MMI) are considered to be dose dependent (Reinwein et al., 1993), especially fatal agranulocytosis and immunosuppressive effects (Ginsberg, 2003; Rivkees et al., 2010; Takata et al., 2009). Besides, radioactive iodine therapy and thyroidectomy may lead to hypothyroidism (Cooper, 2005). Today, due to the problems of common treatments, novel agents that may affect the disease process and treat patients without side effects are needed.

Nature is a valuable reservoir for novel bioactive compounds. Recently, plant‐based therapeutic agents widely used in the prevention and treatment of several diseases (Mushtaq et al., 2018). The extracts prepared from natural sources contain compounds, called secondary metabolites. Secondary metabolites exert biological effects within the organism (Jabeena et al., 2014). In China and some other countries, medicinal plants are used alone or in combination with antithyroid agents for the treatment of hyperthyroidism (Zeng et al., 2007). Our literature review revealed only a few reports on the antithyroid activities of some Brassicaceae plants (Chandra, 2015).

*Descurainia sophia* (L.) Webb ex Prantl (flixweed) is an annual weedy plant, which belongs to the Brassicaceae family. In traditional Iranian manuscripts, it is cited as “Khaksheer,” “Khakshee,” “Todri,” “Khobbah,” and “Bazr‐al‐Khomkhom” (Mirheidar, 2005; Shakeri et al., 2017). This plant is commonly found in Asia, Europe, Africa, and America (Dekić et al., 2017). *Descurainia sophia* has been widely used in traditional medicine in different countries. In traditional Iranian medicine, the seeds of this plant are used to relieve cough, inflammation, and edema. It is also reported as a febrifuge, purgative, and astringent agent (Khan & Wang, 2012; Nimrouzi & Zarshenas, 2016).

Based on previous phytochemical investigations on *D. sophia*, several compounds have been isolated from this plant, such as phenolic compounds (flavonoids and coumarins), fatty acids, lipids, and steroids (HadiNezhad et al., 2015; Mohamed & Mahrous, 2009; Tavakoli & Salarian, 2017). Recent studies have focused on the essential oil and glucosinolates of *D. sophia* (Afsharypuor & Lockwood, 1985; Khodarahmi et al., 2015). Some trace elements, such as iron, zinc, manganese, and copper, also have been identified in *D. sophia* at high levels (Zhongfeng, 2007). It has been reported that the seeds of this plant are rich in some glucosinolates, such as gluconapin (3‐butenyl glucosinolate) (Knight & Stegelmeier, 2007). Glucosinolates are a large group of secondary metabolites which contains sulfur and nitrogen. Plants accumulating glucosinolates possess a thioglucosidase, called myrosinase, which catalyzes the hydrolysis of glucosinolates to many compounds, such as isothiocyanates, nitriles, and thiocyanates (Becker & Juvik, 2016). Also, the glucosinolate hydroxylase enzyme oxidizes unsaturated butenyl in the gluconapin structure, and the cyclization reaction leads to the production of goitrins (Felker et al., 2016).

The production of thyroid hormones requires the micronutrient iodine. Goitrin and thiocyanates inhibit the iodine uptake by the thyroid and can reduce thyroid hormone production (Latté et al., 2011; Tripathi & Mishra, 2007; Vanderpas, 2006).

The sodium iodide symporter on the basolateral part of thyroid follicular cells can inhibit by thiocyanate ion (Dai et al., 1996; Di Bernardo et al., 2011; Tonacchera et al., 2004). Because of the important role of iodine in the synthesis of thyroid hormones and the inhibitory activity of some Brassicaceae plants, such as *D. sophia*, on iodine uptake by the thyroid gland, we hypothesized that it could have beneficial effects on the treatment of hyperthyroidism.

This pilot study aimed to investigate whether *D. sophia* capsules are beneficial in the treatment of hyperthyroidism. This is the first randomized, double‐blind, placebo‐controlled pilot survey on *D. sophia* for the treatment of hyperthyroidism. As well as, the total glucosinolate content of the plant seeds was determined by Ultraviolet–Visible spectrometry.

## 2 MATERIALS AND METHODS

### 2.1 Extract preparation

We collected the ripe seeds of *D. sophia* from Sari, Iran, in May 2018. We have permission to collect *D. sophia* from Mazandaran University of Medical Sciences, Sari, Iran. The plant specimen was identified by Prof Mohammad Azad bakht. The voucher specimen (E1‐26‐261) was deposited at the herbarium of Mazandaran University of Medical Sciences, Sari, Iran. First of all, we crushed the *D. sophia* seeds with an electric grinder at a size that can pass through 350 mesh. In order to have the syrup of the seeds, we used hot methanol extraction based on the ISO 9167‐1 method, with little modification. The methanolic syrup was obtained by mixing the dried samples with hot methanol (70°C) as a solvent and static maceration technique in glass containers. Then, it was incubated at 75°C for 10 min in oven and shaken every 2 min by hand, and after that soaked for 24 h at room temperature. Heating and hot methanol are crucial for deactivating the myrosinase enzyme in the seeds (Doheny‐Adams et al., 2017). Also, we used aluminum foil around the dishes in order to prevent the hydrolyzing of glucosinolate in light. The obtained extract was evaporated to dryness by a rotary evaporator. The output of the extraction was 2.932% (w/w).

### 2.2 Total glucosinolate analysis

The colorimetric analysis of the total glucosinolate content was performed by the method described by Mirzaee et al. (2021). This method was based on the spectrophotometric measurement of glucosinolates (at 420 nm) after alkaline treatment of the plant extract (for producing 1‐thioglucose) and chromogenic reaction of 1‐thioglucose with the ferricyanide reagent (for reducing it). Briefly, 7.5 mL of acetate buffer (pH = 4.2, 0.2 M) was added to 500 mg of powdered seeds and placed in a boiling bath for 15 min. The syrup was chilled and mixed with 1.5 mL of barium and lead acetate solution (0.5 M). Next, 0.4 g of polyvinylpolypyrrolidone was added to the blend and stirred for 15 min. Eventually, it was centrifuged at 10,000 rpm for 5 min by adding 1.5 mL of sodium sulfate solution (2 M).

For the alkaline treatment of glucosinolates, the supernatant was mixed with an equal volume of 2 M sodium hydroxide solution and neutralized by hydrochloric acid (37%). The mixture was centrifuged at 10,000 rpm for 3 min, and the supernatant was mixed with an equal volume of 2 mM ferricyanide. The absorbance was measured rapidly against the blank solution. Different concentrations (0–500 μL) of sinigrin stock solution (5 mg of sinigrin in 1 mL of distilled water) were prepared as described above (alkaline treatment) and used to create the standard calibration curve. The glucosinolate content was used as the standard value in the *D. sophia* capsules.

### 2.3 Preparation of capsules

The seed extract was dissolved in water at 1:2 ratios, and then, the required amount of Aerosil was added. The required firm gelatin capsules (size 0) were packed with powdered *D. sophia* syrup and placebo (containing all components of the capsule formulation, except *D. sophia* syrup) individually, we placed the two capsules indifferent plastic containers with the same shape and color. Generally, the ordinary dose of *D. sophia* seeds, relying on the World Health Organization (WHO) and traditional Iranian medicine (Hosseini et al., 2018), is 350 mg at maximum. Therefore, this dose was used in our survey. Sixty capsules were placed in each labeled bottle. A six‐digit number was labeled on each bottle by the principal investigator. These codes were saved in a locked file on the computer and the patients, the physician, and the investigator of clinical responses were not aware of the type of intervention. All data were collected by a single investigator. At the end of the survey, the principal investigator decoded the numbers and attributed each of them to the proper groups. Our formulations were accomplished under sterile conditions.

### 2.4 Evaluating parameters of capsules

#### 2.4.1 Angle of repose

The angle of repose (*α*) was defined by using the funnel way. The powder‐dried form of the seeds flowed through a funnel that could be raised vertically until the maximum cone height (*h*) was gained. We measured the radius of the heap (*r*), and then calculated angle of repose (*α* = Arctan [*h*/*r*]) (Martin, 1983).

#### 2.4.2 Weight variations

Weight variation was evaluated to get the assurance that each capsule had the right amount of the drug. The test was performed by weighing 20 capsules individually using the analytical balance, then we calculated the average weight of them, and compared each tablet's weight with the average (Indian Pharmacopoeia Commission, 2014).

#### 2.4.3 Disintegration time

We put one capsule in each of the six tubes in the synthesis, and the synthesis was suspended in water. Then, we added discs to each tube, and the temperature was kept at 37 ± 2°C (United States Pharmacopoeia, 2005).

#### 2.4.4 Stability study

The stability study, according to the International Council on Harmonization (ICH) guidelines, was performed for 1 month at 40°C ± 2°C/75% RH ± 5%. The formulation was evaluated in terms of disintegration time. Organoleptic testing, involving the assessment of flavor, odor, appearance, and color of *D. sophia* seed extract powder, was performed at the beginning of the study and every month.

#### 2.4.5 Inclusion and exclusion criteria

The inclusion criteria were described below: (1) untreated hyperthyroidism; the diagnosis of hyperthyroidism was based on the presence of high serum free thyroxine (FT4) (≥1.8–7.7 ng/mL or 23–100 pmol/L) and/or free triiodothyronine (FT3) (≥5.7 pg/mL) and low serum thyroid‐stimulating hormone (TSH) (≤0.4 mU/L); (2) informed consent and cooperation with regular follow‐ups; (3) absence of exclusion criteria; and (4) being in the age range of 20–60 years. The exclusion criteria were described below: (1) lack of informed consent; (2) comorbidities, such as diabetes mellitus, ischemic heart disease, liver or kidney failure, alcoholism, and smoking; (3) pregnancy or lactation; and (4) baseline alanine aminotransferase twice higher than the normal upper limit.

#### 2.4.6 Criteria for discontinuation of patient's cooperation

The exclusion criteria were as follows: (1) not experiencing any adverse drug reactions during the study but should stop intervention for other reasons like other disease; (2) they stopped the trial because they thought the drugs to be ineffective; (3) they had dangerous adverse effects during trial; (4) they could not continue the treatment (patient or family's request for drug withdrawal); (5) they had exacerbation of the disease, and (6) they had allergy to the test drugs (Takakuwa & Kina, 2014).

#### 2.4.7 Exclusion criteria during the study

The trial would be obsolete if (1) serious opposite events happened; or (2) the patients developed allergies.

#### 2.4.8 Sample size and randomization

**FIGURE 1:**
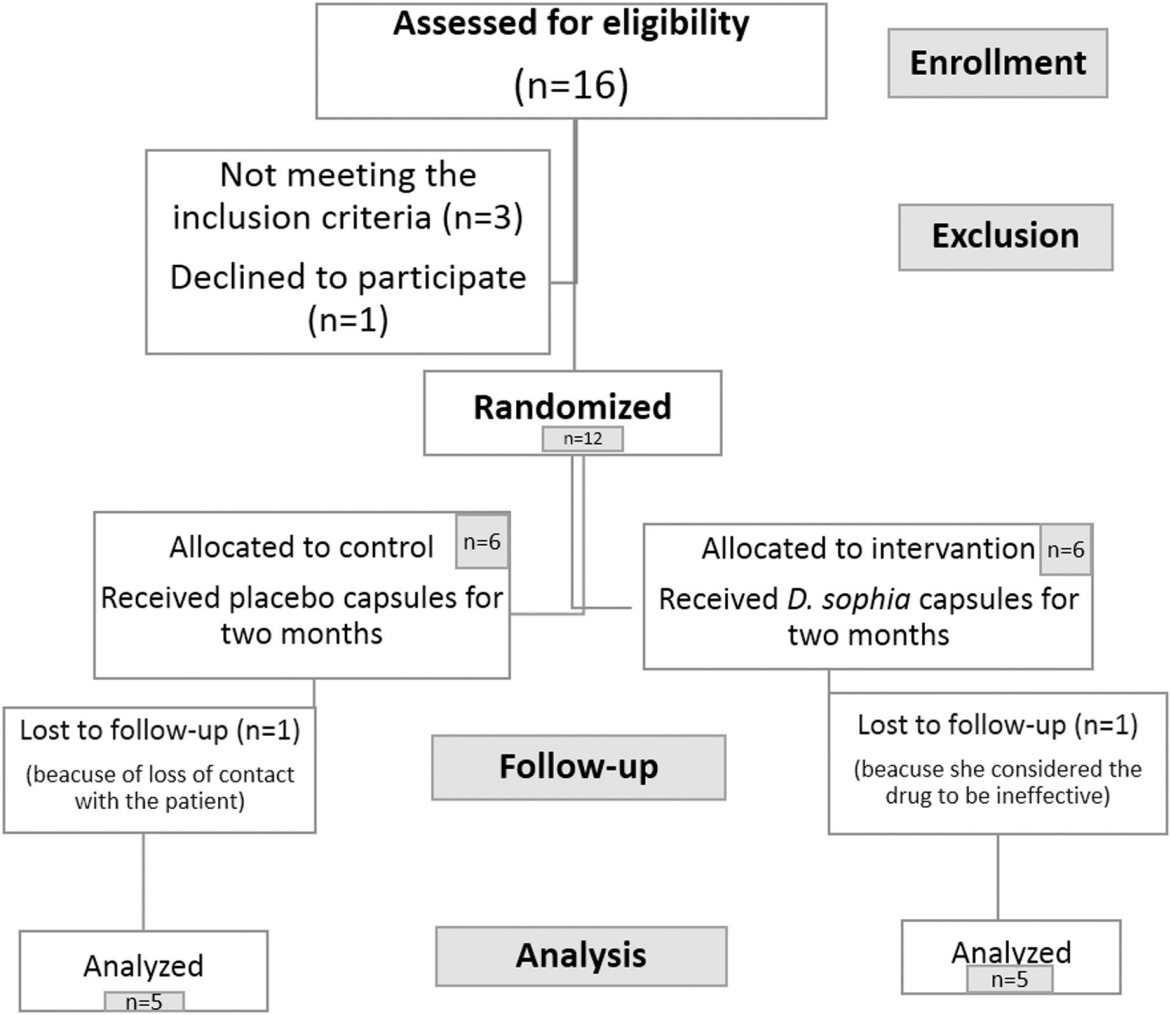
The flow diagram of the study.

Due to our search, no practical action has been taken to use this extract for humans. With an effect size of 0.7, confidence interval of 95%, and test power of 85%, for the calculation of sample size, 10 newly hyperthyroid female patients were selected among eligible outpatients, who were treated at the Department of Endocrinology of Touba Clinic in Sari, Iran from May 2019 to July 2020. All the patients received an oral explanation about the study by a trained assistant. The patients were free to participate in the study voluntarily. Also, we obtained written informed consent from all participants who accepted to be a part of our trial. They were randomly divided into two groups by using a random number table in software (five patients per group) (Figure 1).

#### 2.4.9 Study intervention, outcomes, and follow‐up

The body mass index (BMI (weight (kg)/[height (m)]^2^)) was calculated for all patients after measuring their height and weight in the clinic.

Patients in both groups were treated with methimazole at 15 mg/day to relieve the symptoms, because this is the first study on *D. sophia* for the treatment of hyperthyroidism in humans, and it is not a well‐accepted treatment for hyperthyroidism. Treatment with MMI continued during the study (for 2 months). A similarly labeled bottle, containing *D. sophia* or placebo, was then distributed among the patients. The patients were instructed to use capsules for 2 months (one capsule twice daily). Patients received 350 mg of dried *D. sophia* seeds per day. One week after the onset of the study, we telephoned the patients to follow possible side effects like constipation or diarrhea or headache etc., or any other problems, which were recorded by the investigator. Also, we set monthly visits for the patients. All data were collected by one investigator. Also, we gave phone number to the patients, so that they could call us if they had any questions.

The analytical test indices, including FT3, FT4, and TSH, were measured after collecting blood samples from patients according to reported methods before the onset of trial and after 60 days, respectively. Also, BMI, age, and family history of hyperthyroidism were recorded before the onset of treatment. The main symptoms of the disease, including nervousness and irritability, insomnia, eye problems (dry eye syndrome or blurred vision), dyspnea, heat intolerance, and tremor, were recorded once before the onset of treatment and again after 30 and 60 days of treatment, respectively. For each symptom, a score of 1–5 was assigned, as shown in Table 1. This 5‐point scale can help us to quantify symptoms (Benvenga et al., 2001). Also, we asked the patients to bring a bottle of capsules for monthly visits to assess the Compliance. We asked about possible adverse events in all visits and phone calls. All methods were conducted in accordance with the guidelines.

**TABLE 1:** Standardized evaluation of the symptomatology.

	Scores
Absent	1
Occasional, but disturbing when present	2
Frequent	3
More frequent	4
Constant/intolerable	5

### 2.5 Statistical analysis

Results are offered as mean ± SD (standard deviation). SPSS version 23 was used. *p* < .05 was set as statistical significance. The normal distribution of data was determined using Shapiro–Wilk test. First of all, the baseline features of the two groups were compared. Mean hormone concentration between groups was contrasted using nonparametric Mann–Whitney test for nonparametric data. Changes in symptom score with time were compared using analysis of variance and repeated measurement. To contrast the mean scores before and after the treatment in both groups, paired *t*‐test or its nonparametric equivalent, Wilcoxon signed‐rank test, was used (Takakuwa & Kina, 2014).

## 3 RESULTS

### 3.1 Total glucosinolate content

**FIGURE 2:**
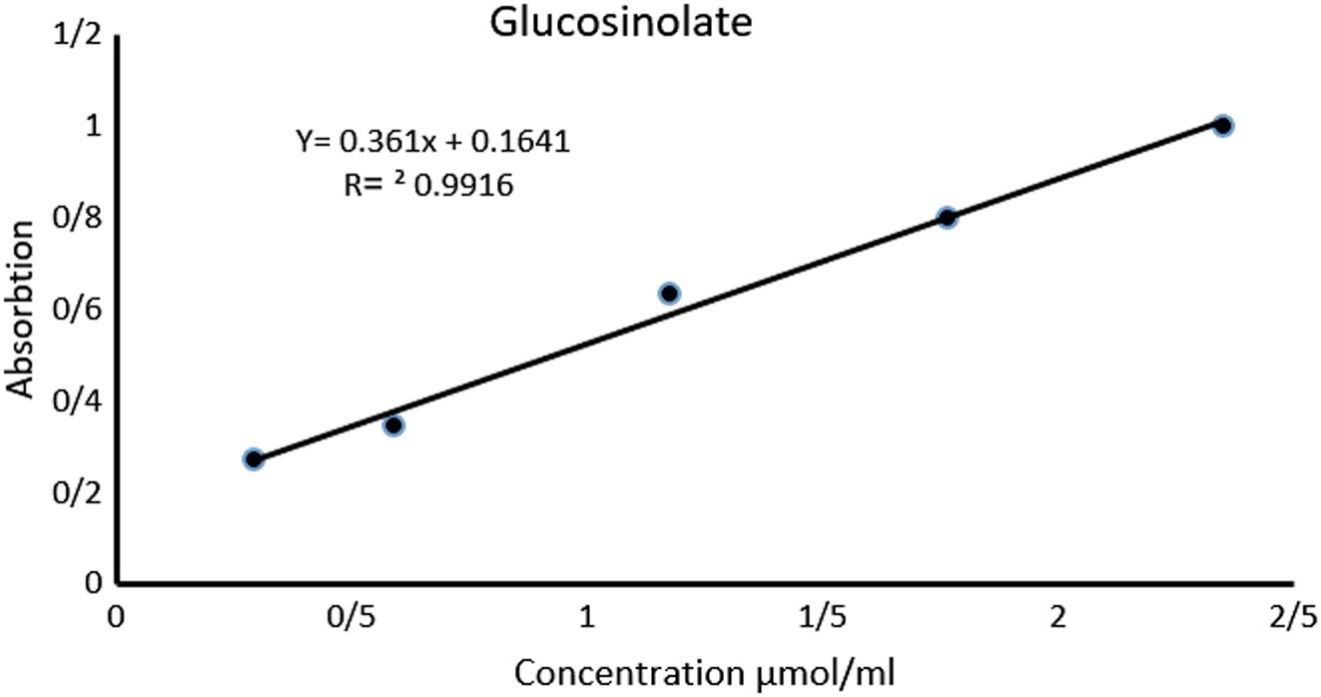
The standard curve of sinigrin.

The total glucosinolate content was determined 9.59 ± 0.4 mg sinigrin equivalents per gram of dried seeds (9.59 mg SIN/g), based on the following standard curve (Figure 2).

### 3.2 Physicochemical parameters of capsules

The angle of repose was found to be 26.60° ± 0.122°. The average weight of the capsules was 0.35 g. The disintegration time for the hard gelatin capsule was 5.05 ± 0.816 min. Stability study showed no significant change in the disintegration time and organoleptic properties, such as flavor, odor, appearance, and color of the formulation.

### 3.3 Demographic data

The flowchart of participant enrollment is presented in Figure 1. After decoding the questionnaires, it was found that five patients had received the placebo, and five patients had been treated with *D. sophia* (*DS* group); we did not discover any significant difference between the two groups in terms of demographic characteristics. Changes in age and BMI are shown in Table 2. The mean age of the subjects was 32.06 ± 8.64 and 32.13 ± 8.8 years in the *DS* and placebo groups, respectively (*p* = .34). The BMI was 26.85 ± 4.5 and 26.12 ± 3.5 kg/m^2^ in the *DS* and placebo groups, respectively (*p* = .14). All patients were weighed using the same scale. All patients in both groups lived in urban regions. One patient from each group had a family history of thyroid gland dysfunction.

**TABLE 2:** The age and BMI of the study groups.

	Placebo	*DS*	*p*‐value
Mean age	32.13 ± 8.8	32.06 ± 8.64	.34
BMI (kg/m^2^)	26.12 ± 3.5	26.85 ± 4.5	.14

*Note*: Numerical values are reported as mean ± SD.

Abbreviations: BMI, body mass index; *DS*, *Descurainia sophia*.

### 3.4 Thyroid hormones

Changes in the serum FT3, FT4, and TSH levels at baseline and at the end of the trial are reported in Table 3. The serum level of FT3 at baseline was 9.4 ± 5.9 Pmol/L in the *DS* group and 9.3 ± 5.02 Pmol/L in the placebo group; the serum level of FT4 was 31.77 ± 3.7 Pmol/L in the *DS* group and 32.46 ± 3.4 Pmol/L in the placebo group; and the serum level of TSH was 0.05 ± 0.02 Pmol/L in the *DS* group and 0.051 ± 0.01 Pmol/L in the placebo group. Differences between the groups regarding FT3, FT4, and TSH were not significant (*p* > .05). Administration of the extract significantly improved the TSH level (4.7 ± 0.1 μIU/mL) in the *DS* group as compared to the placebo group. The serum FT3 level in the *DS* group significantly reduced (5.9 ± 2.5 Pmol/L) in comparison with the placebo group (6.9 ± 5.3.3 Pmol/L). Also, the serum FT4 level was significantly reduced in the *DS* group (29.1 ± 3.1 Pmol/L) as compared to the placebo group (39.07 ± 7.3 Pmol/L).

**TABLE 3:** Serum FT3, FT4, and TSH levels of participants at study before and after treatment.

Variables	Groups		*p*‐value
	*DS*	Placebo	
Serum TSH (μIU/mL)			
Before treatment	0.05 ± 0.02	0.051 ± 0.01	.55
End of treatment	4.7 ± 0.1	3.7 ± 0.3	*p* < .05
Serum FT4 (Pmol/L)			
Before treatment	31.77 ± 3.7	32.64 ± 3.4	.29
End of treatment	29.1 ± 3.1	39.77 ± 7.3	*p* < .05
Serum FT3 (Pmol/L)			
Before treatment	9.4 ± 5.9	9.3 ± 5.02	.74
End of treatment	5.9 ± 2.5	6.9 ± 5.3	*p* < .05

*Note*: Numerical values are reported as mean ± SD.

Abbreviation: *DS*, *Descurainia sophia*.

### 3.5 Effect of *D. sophia* capsules on the symptoms

**FIGURE 3:**
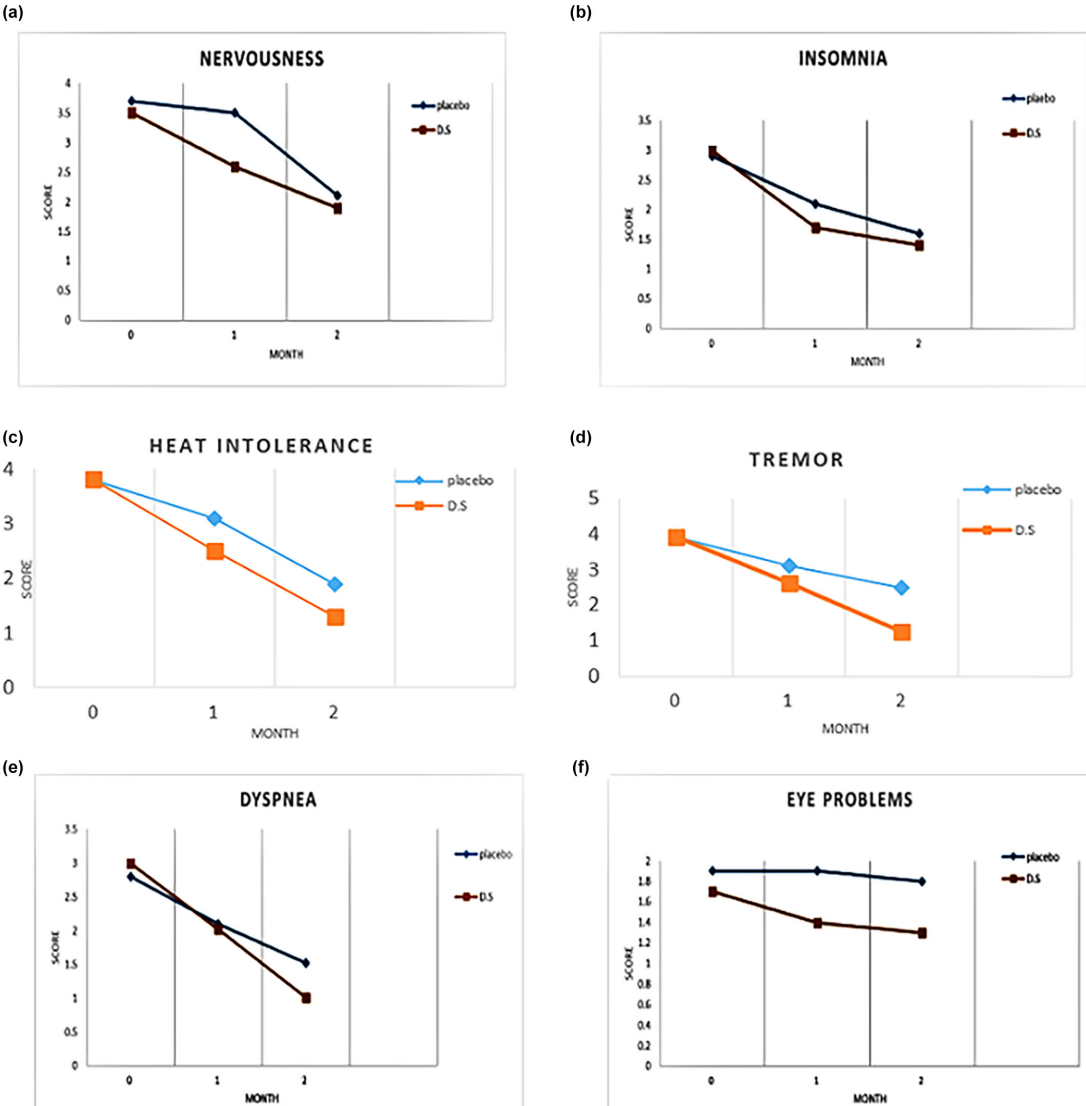
Nervousness trend of changes during 2 months (a), Insomnia trend of changes during 2 months (b), Heat intolerance trend of changes during 2 months (c), Tremor changes (d), Dyspnea (e), Eye problems (f), *DS*, *Descurainia sophia*.

Changes in the symptoms, such as nervousness, insomnia, eye problems, dyspnea, heat intolerance, and tremor at baseline and in the first and second months of the trial, are reported in Table 4. The results showed that the baseline features of the two groups did not differ. There was a significant improvement in the symptoms (*DS* group vs. placebo group) at the end of treatment in terms of nervousness (mean score: 1.9 ± 0.1 vs. 2.1 ± 0.1), insomnia (mean score: 1.4 ± 0.1 vs. 1.6 ± 0.1), heat intolerance (mean score: 1.3 ± 0.1 vs. 1.9 ± 0.1), tremor (mean score: 1.25 ± 0.1 vs. 2.5 ± 0.1), dyspnea (mean score: 1.01 ± 0.02 vs. 2.52 ± 0.01), and eye problems (mean score: 1.3 ± 0.1vs. 1.8 ± 0.1) (*p* < .05) (Figure 3).

**TABLE 4:** Effect of *D. sophia* capsules on the thyrotoxic symptoms.

Variables	Groups		*p*‐value
	*DS*	Placebo	
Nervousness			
Before treatment	3.5 ± 0.1	3.7 ± 0.1	.23
In the first month	2.6 ± 0.1	3.5 ± 0.1	*p* < .05
In the second month	1.9 ± 0.1	2.1 ± 0.1	*p* < .05
Insomnia			
Before treatment	2.99 ± 0.1	2.9 ± 0.2	.777
In the first month	1.7 ± 0.1	2.1 ± 0.1	*p* < .05
In the second month	1.4 ± 0.1	1.6 ± 0.1	*p* < .05
Heat intolerance			
Before treatment	3.8 ± 0.1	3.8 ± 0.1	.812
In the first month	2.5 ± 0.1	3.1 ± 0.2	*p* < .05
In the second month	1.3 ± 0.1	1.9 ± 0.1	*p* < .05
Tremor			
Before treatment	3.5 ± 0.1	3.9 ± 0.2	.415
In the first month	2.6 ± 0.1	3.1 ± 0.1	*p* < .05
In the second month	1.25 ± 0.1	2.5 ± 0.1	*p* < .05
Dyspnea			
Before treatment	2.99 ± 0.1	2.8 ± 0.1	.627
In the first month	2.02 ± 0.01	2.1 ± 0.2	*p* < .05
In the second month	1.01 ± 0.02	2.52 ± 0.01	*p* < .05
Eye problems			
Before treatment	1.7 ± 0.1	1.9 ± 0.1	.249
In the first month	1.4 ± 0.1	1.9 ± 0.1	*p* < .05
In the second month	1.3 ± 0.1	1.8 ± 0.1	*p* < .05

Abbreviation: *DS*, *Descurainia sophia*.

## 4 DISCUSSION

Our study was a double‐blind, placebo‐controlled pilot clinical trial that reported good results regarding the effectiveness of *D. sophia* capsules at a dose of 350 mg/day as an adjuvant treatment of hyperthyroidism. Based on our results, the clinical symptoms of the disease, including nervousness and irritability, insomnia, eye problems, dyspnea, heat intolerance, and tremor, were significantly reduced in the *DS* group in comparison with the placebo group. And, the level of TSH increased significantly in the DS group as compared to the placebo. A significant reduction was detected in the concentrations of FT3 and FT4 hormones in the *DS* group in comparison with the placebo.

Moreover, we investigated the total glucosinolate content of plant seeds based on the sinigrin standard curve. The total glucosinolate content was calculated to be 9.59 mg SIN/g. In comparison with a previous study (Montazeri et al., 2020), the level of glucosinolates in other Brassicaceae plants, such as *Lepidium perfoliatum* seeds (7.71 mg SIN/g), was lower than our finding. The glucosinolates exerted an interesting effect on hyperthyroidism. Based on a study by Khan and Wang (2012), the glucosinolate constituents of *D. sophia* seeds possess goitrogenic effects. They found that the plant seeds contained high concentrations of some goitrogenic glucosinolates, such as gluconapin (3‐butenyl glucosinolate).

It has been reported that the glucosinolate hydroxylase enzyme oxidizes unsaturated butenyl in the gluconapin structure, and the cyclization reaction leads to the production of goitrin.

Goitrin showed to inhibit the production of thyroid hormones by decreasing the iodine uptake into the thyroid gland. Based on a study by Felker et al. (2016), the huge amount (e.g., >1 kg/d for several months) of some Brassicaceae plants with high progoitrin concentrations is associated with the potential risk of hypothyroidism. Moreover, Kelley and Bjeldanes (1995) found that a diet of progoitrin (154 μmol/100 g) can cause hypothyroid state in the rats. Moreover, Langer et al. (1971) showed that 25 mg (194 μmol) was the minimal amount of goitrin needed to reduce the uptake of radioiodine; in contrast, a smaller ingested amount (i.e., 10 mg or 70 μmol) had no effect on the uptake of it in rats and men.

Thiocyanate ion, which is manufactured by the destruction of indole glucosinolates, is another important substance with probable inhibitory effects on the thyroid gland. Based on a study by Tonachra et al. (Jezek et al., 1999), this compound can inhibit the radioactive iodide uptake by sodium–iodide symporter. The sodium/iodide symporter (NIS or SLC5A5) is involved in iodide uptake by thyroid follicular cells. It is an important part of iodine metabolism. Overall, the available treatments for hyperthyroidism, such as antithyroid drugs, thyroidectomy, and radioactive iodine, can cause some unwanted effects. In this study, we took a further step toward the treatment of hyperthyroidism. This is the first evaluation of the effect of *D. sophia* seeds on hyperthyroidism in humans. Although we do not know their exact mechanism of action till now, goitrin and thiocyanate ion may be important in the inhibition of the thyroid gland.

Our study had some limitations. First, the low number of patients meeting the inclusion criteria led to the small sample size, which is one of the most important limitations of this study. Second, lack of previous studies in this area is another shortcoming. Third, we faced some problems in data collection; we had some questions about the symptoms, and the patients were required to choose a number to describe the severity of their problem. Fourth, we could not have an accurate monitoring of drug use by the patients (monitoring was based on the patient's report). Finally, nonacceptance of the drug by the patient due to lack of information and assurance of its content was another limitation of this study.

## 5 CONCLUSIONS

In conclusion, our findings showed that the methanolic extract of *D. sophia* can be useful as an adjunctive treatment for hyperthyroidism, but further clinical trials are necessary to show exact benefits, especially considering the fact that the sample size of this study was not powered enough.

## AUTHOR CONTRIBUTIONS

**Fatemeh Farzameh:** Conceptualization (equal); investigation (equal); methodology (equal); supervision (equal); writing – original draft (equal); writing – review and editing (equal). **Mohammad Azadbakht:** Conceptualization (equal); investigation (equal); methodology (equal); supervision (equal); writing – original draft (equal); writing – review and editing (equal). **Zahra Kashi:** Conceptualization (equal); investigation (equal); methodology (equal); supervision (equal); writing – original draft (equal); writing – review and editing (equal). **Hossein Asgarirad:** Methodology (equal); project administration (equal); writing – original draft (equal); writing – review and editing (equal). **Ebrahim Salehifar:** Conceptualization (equal); investigation (equal); methodology (equal); supervision (equal); writing – original draft (equal); writing – review and editing (equal). **Fatemeh Mirzaee:** Formal analysis (equal); methodology (equal); writing – original draft (equal); writing – review and editing (equal). **Ali Davoodi:** Methodology (equal); project administration (equal); writing – original draft (equal); writing – review and editing (equal). **Shervin Amirkhanloo:** Methodology (equal); project administration (equal); writing – original draft (equal); writing – review and editing (equal).

## FUNDING INFORMATION

This study has been supported by Mazandaran University of Medical Sciences (Grant No. 1789).

## CONFLICT OF INTEREST STATEMENT

The authors declare that they do not have any conflict of interest.

## ETHICS STATEMENT

Our study was a randomized, double‐blind, placebo‐controlled pilot trial planned to compare *Descurainia sophia* with placebo capsules. After obtaining approval from the Ethical Committee of Mazandaran University of Medical Sciences (Ethics Code: IR.MAZUMS.REC.1398.360), the study proposal was approved by the Iranian Registry of Clinical Trials (IRCT) with a registry code of IRCT20130911014630N6 (Registration date: 26/08/2019). Written informed consent was obtained from all study participants.

## Data Availability

The datasets generated during and/or analyzed during the current study are available from the corresponding author on reasonable request.
